# Computational Screening Strategy for Drug Repurposing Identified Niclosamide as Inhibitor of Vascular Calcification

**DOI:** 10.3389/fcvm.2021.826529

**Published:** 2022-01-20

**Authors:** Takeshi Tanaka, Takaharu Asano, Takehito Okui, Shiori Kuraoka, Sasha A. Singh, Masanori Aikawa, Elena Aikawa

**Affiliations:** ^1^Center for Interdisciplinary Cardiovascular Sciences, Brigham and Women's Hospital and Harvard Medical School, Boston, MA, United States; ^2^Center for Excellence in Vascular Biology, Cardiovascular Medicine, Brigham and Women's Hospital and Harvard Medical School, Boston, MA, United States; ^3^Department of Human Pathology, Sechenov First Moscow State Medical University, Moscow, Russia

**Keywords:** calcification, drug discovery, drug repurposing, Wnt signaling, mouse models, proteomics

## Abstract

Vascular calcification is a cardiovascular disorder with no therapeutic options. We recently reported that *o*-octanoyltransferase (CROT) suppression can inhibit vascular calcification *in vivo* and *in vitro* through amelioration of mitochondrial function and fatty acid metabolism. Inhibiting calcification with a small molecule compound targeting CROT-associated mechanisms will be a promising non-invasive treatment of vascular calcification. Here we used a computational approach to search for existing drugs that can inhibit vascular calcification through the CROT pathway. For screening of the compounds that reduce CROT expression, we utilized the Connectivity Map encompassing the L1000 computational platform that contains transcription profiles of various cell lines and perturbagens including small molecules. Small molecules (*n* = 13) were identified and tested in human primary smooth muscle cells cultured in osteogenic media to induce calcification. Niclosamide, an FDA-improved anthelmintic drug, markedly inhibited calcification along with reduced alkaline phosphatase activity and *CROT* mRNA expression. To validate this compound *in vivo*, LDL receptor (*Ldlr*)-deficient mice fed a high fat diet were given oral doses of niclosamide (0 or 750 ppm admixed with diet) for 10 weeks. Niclosamide treatment decreased aortic and carotid artery calcification as determined by optical near infrared molecular imaging (OsteoSense680) and histological analysis. In addition, niclosamide improved features of fatty liver, including decreased cholesterol levels along with decreased Crot expression, while plasma total cholesterol levels did not change. Proteomic analysis of aortic samples demonstrated that niclosamide affected wingless/integrated (Wnt) signaling pathway and decreased runt-related transcription factor 2 (Runx2) expression, an essential factor for calcification. Our target discovery strategy using a genetic perturbation database with existing drugs identified niclosamide, that in turn inhibited calcification *in vivo* and *in vitro*, indicating its potential for the treatment of vascular calcification.

## Introduction

Cardiovascular disease is a primary cause of death globally. Vascular calcification, characterized as deposition of hydroxyapatite in the arterial wall, is a major contributor to cardiovascular disease (1, 2). Clinical evidence associated vascular calcification with atherosclerosis, diabetes and kidney disfunction. Despite the efforts that tested a variety of therapeutic options to prevent cardiovascular calcification (3), effective drug therapies remain unavailable.

We recently reported carnitine *o*-octanoyltransferase (CROT) as a candidate target to suppress vascular calcification (4). Proteomics of human smooth muscle cells (hSMCs) identified elevated levels of CROT during the transition to a procalcifying phenotype. Small interfering RNA (siRNA)-mediated suppression of *CROT* in hSMCs and genetic deletion of *Crot* in mice inhibited vascular calcification. Additional proteomics, lipidomics and network analysis studies deduced that CROT inhibition suppresses calcification potential by ameliorating mitochondrial function and fatty acid metabolism (4). Although this recent study suggests CROT as a promising target, it is only the beginning of multiple steps toward confirming CROT inhibition is indeed a viable therapy for cardiovascular calcification. Small molecule or compound inhibitor screens typically follow the target discovery stage; however, conventional screening assays face many challenges including escalating costs and excessive amount of time required for drug development (5).

Conventional approaches employ high-throughput compound-protein physical screening assays. It is estimated that less than a dollar will be returned for every dollar spent on this type of research and development (6). To mitigate increasing costs to develop new compounds, drug repositioning, a strategy to identify novel uses for approved drugs with new pharmaceutical indications, is increasing in popularity. Drug repurposing offers several advantages compared to conventional approaches, including lower risk of failure because safety profiles of approved drugs have been already established. Importantly, the time frame required for drug development can be shorter, since most preclinical studies (e.g., medicinal pharmacology, toxicology profiles, formulation development) would have been completed. Yet, newer alternatives exist to identify candidate compounds, such as virtual approaches (e.g., signature matching, genetic perturbation screens, molecular docking, retrospective clinical analysis) that can be coupled to traditional binding and phenotypic screening (7, 8).

Given that many of these virtual approaches are publicly available, we sought to identify candidate compounds that can potentially reduce CROT expression, by screening a genetic perturbation resource known as L1000, using the Connectivity Map (CMap) (9, 10). Since the reduction of *CROT* mRNA inhibits calcification (4), we screened for small molecules that were predicted to reduce *CROT* expression, with the idea that they could be candidate calcification inhibitors. We then validated them *in vitro* and *in vivo*. Among the tested candidate compounds, niclosamide, a Food and Drug Administration (FDA)-approved anthelmintic drug (11, 12), inhibited calcification in both human SMCs and in atherosclerotic lesions of *Ldlr*^−/−^ mice.

Overall, this present study reports the identification of a candidate small molecule inhibitor of *CROT* expression through a computational approach, that in turn was demonstrated to inhibit calcification *in vitro* and *in vivo*. More specifically, our strategy demonstrates that repurposing a drug already used in clinic is a viable option for the treatment of vascular calcification.

## Materials and Methods

Additional detailed materials and methods are included in Supplementary Materials.

### Compound Selection With CMap Using L1000 Platform

Level 5 gene expression profiles in the L1000 dataset were downloaded from Gene Expression Omnibus website (Accession No.; GSE92742). This level 5 dataset provides the most robust differential expression values consisting of over 10,000 genes as described below, and contains 473,647 profiles induced by exposing 76 cell types to 51,219 perturbagens. The expression profile comprises expression levels that were compared to the controls (the background of the plate). Each perturbagen's expression profile comprises 12,328 genes, 978 of which are measured directly (called landmark genes) (10). Of the remaining genes, 9,196 were well-inferred (i.e., their expression levels correlate to the actual measured levels with *p*-values < 0.05); and the other 2,154 were less-well inferred genes. CROT was among the well-inferred genes. The gene expression profiles treated with small molecules were extracted by setting “trt_cp” as the perturbagen type, resulting in 205,033 profiles using 20,412 small molecules that are distinguished by perturbagen ID. Mean expression levels of CROT and the *p*-values comparing to the control were calculated for each small molecule that is used for at least five gene profiles. We then selected small molecules that decrease CROT expression (i.e., cause the negative expression levels) with *p*-value < 1 × 10^−6^.

### Cell Culture and Osteogenic Induction for Compound Screening and Validation

Human coronary artery smooth muscle cells (hSMCs, C-12511, PromoCell) were cultured in SMC growth medium (C-22052, PromoCell) at 37°C with 5% CO_2_. Cells were inoculated on 24 or 48 well plates by 1 x 10^6^ cells/ml with normal condition medium (NM, DMEM with 4.5 g/L glucose (10569010, Thermo Fisher Scientific containing 10% fetal bovine serum). After 24-h, medium was changed to osteogenic medium (OM, NM added with 10% fetal bovine serum, 10 mmol/L β-glycerophosphate disodium salt pentahydrate, 100 μmol/L L-ascorbic acid 2-phosphate sesquimagnesium salt hydrate, 10 nmol/L dexamethasone). Each compound then was added in formulation of DMSO solution in 1-to-2,000-fold dilution. DMSO was used as control with NM and OM. Media containing compound was changed every 3-4 days.

### *In vivo* Validation Study Using *Ldlr*-Deficient Mice

Eight to 10-week-old male *Ldlr*-deficient (*Ldlr*^−/−^) mice (Cat No. 002207, Jackson Laboratories) were fed a high-fat and high-cholesterol diet (HFD; D12108CO, Research diet Inc) for 15 weeks to induce atherosclerosis and cardiovascular calcification. Niclosamide (N3510, Sigma Aldrich Co.) was mixed into HFD at 750 ppm concentration. The dose and duration for Niclosamide treatment were determined based on the results from our pilot study, which showed 750 ppm treatment for 8 weeks after 7 weeks of HFD suppressed vascular calcification, while 250 ppm of Niclosamide did not show substantial changes (Supplementary Figures IA,B). Mice were randomly assigned into three groups of 15-17 animals per group: (1) normal chow diet, (2) HFD and (3) HFD containing Niclosamide for 10 weeks after 5 weeks of HFD treatment (Figure 2A). All animal experiments were approved by and performed in compliance with Beth Israel Deaconess Medical Center's Institutional Animal Care and Use Committee (protocol no. 010-2016).

### Mass Spectrometry Data

All mass spectrometry and resulting search data have been deposited to the ProteomeXchange Consortium via the PRIDE partner repository21 with the data set identifier PXD029963.

### Statistical Analysis

ANOVA filtering followed by Dunnett, or Tukey multiple comparison test or Student *t*-tests were performed by Prism 8 software (GraphPad software). The normality and variance were tested to determine whether the applied parametric tests were appropriate. Group comparison in proteomics data analysis was made using ANOVA filtering by Qlucore Omics Explorer (Qlucore AB).

## Results

### Compound Selection With CMap Using L1000 Platform

**Figure 1:**
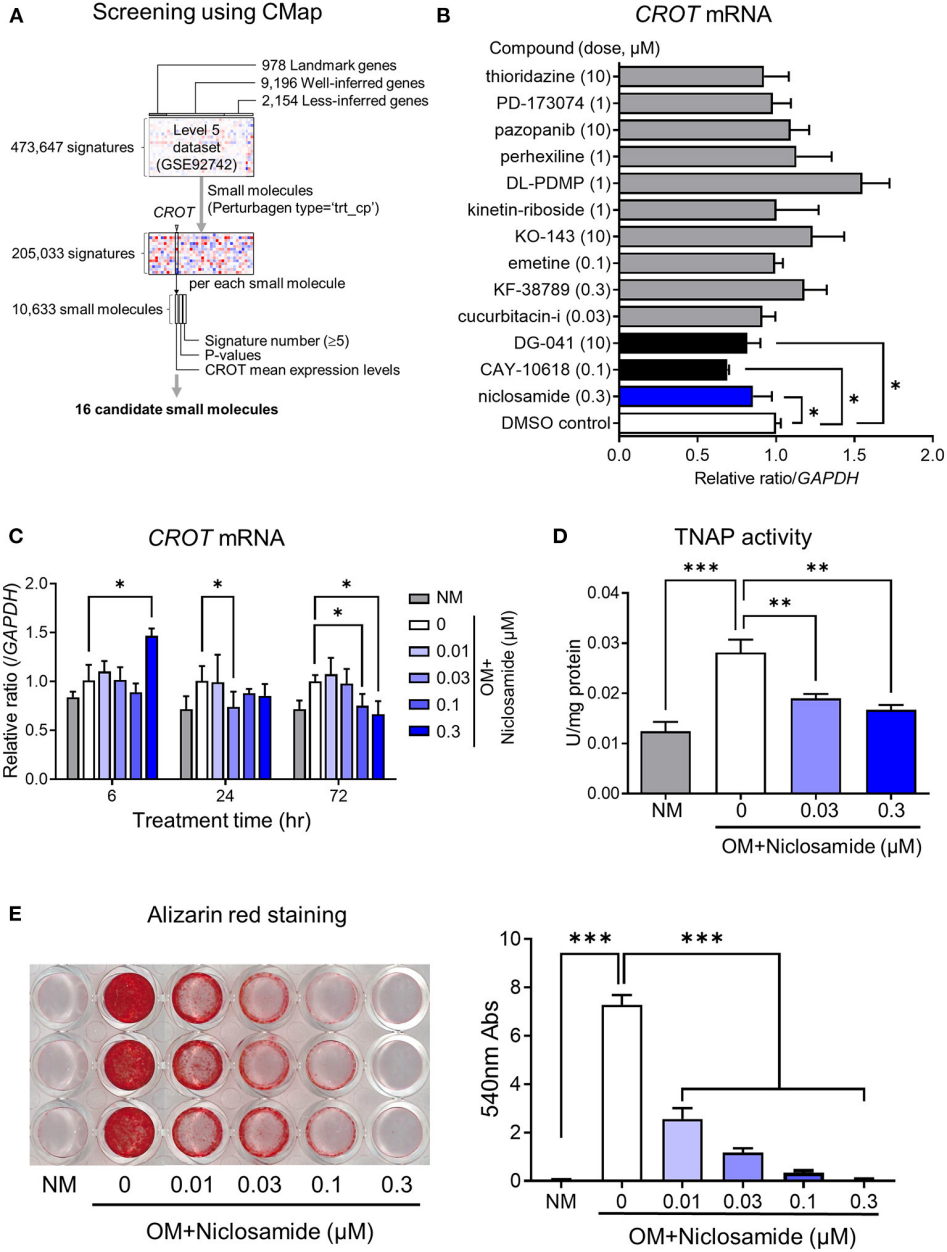
*In silico* screening and *in vitro* validation of candidate small molecule inhibitors of *CROT* expression and calcification in hSMCs. **(A)** Flowchart of candidate small molecule selection using connectivity map (CMap) with L1000 database. Small molecules “pert_type” = “trt_cp” extracted from all perturbagens in L1000. **(B)** The effects of candidate compounds on *CROT* (carnitine O-octanoyltransferase) transcript levels in hSMCs cultured in osteogenic media. The μM concentrations are listed. **(C)** *CROT* transcript levels in SMCs at 6-, 24-, and 72-h treatment with niclosamide. **(D)** TNAP (tissue-non-specific alkaline phosphatase) activity in hSMCs treated with niclosamide for 14 days. **(E)** Alizarin red staining and quantification in hSMCs treated with Niclosamide for 21 days. Data were analyzed by ANOVA with Dunnett's multiple comparison test, *P < 0.05, **P < 0.01, ***P < 0.001. **(C,D)** Each panel present data from the same donor, with three technical replicates. Error bars are mean ± SD.

To identify promising candidate compounds that can reduce *CROT* expression, we turned to a computational screening approach using CMap within L1000 platform (Figure 1A). First, 16 candidate compounds were selected as shown in Table 1. Among these candidates, IKK-2-inhibitor-V was excluded from further consideration since IKKβ-deficient mice showed increased vascular calcification (13). Mebendazole and D-64131, microtubule destabilizing compounds, were also excluded because microtubule stabilization has been already shown to attenuate calcification via inhibition of osteogenic signaling (14). The remaining 13 candidates were then assessed for their ability to decrease *CROT* mRNA expression and suppress calcium deposition in hSMCs cultured in osteogenic media (OM). This osteogenic condition increased *CROT* transcriptional levels in hSMCs as previously reported (4). The maximum concentration was determined based on each compound's solubility and cell toxicity (Supplementary Figure IIA). Of these 13 compounds, niclosamide (0.3 μM), CAY-10618, and DG-041 decreased *CROT* mRNA expression in hSMCs cultured in OM after 72 h as compared to DMSO control (Figure 1B). Alizarin red staining after 21-day stimulation with OM showed that PD173074, pazopanib, perhexiline, and niclosamide decreased calcium deposition compared to control. Only niclosamide, however, decreased both *CROT* mRNA expression and calcium deposition, notably without morphological cell damage (Supplementary Figure IIA), in hSMCs cultured in osteogenic media. No previous studies reported niclosamide as a calcification inhibitor. These results led us to pursue this compound further.

**Table 1:** Candidate small molecules that possibly decrease CROT expression.

**Ranking**	**pert_id**	**pert_iname**	**CROT** **(mean expression)**	***P*-value**
1	BRD-A28105619	cucurbitacin-i	−0.953	4.39E-14
2	BRD-A54927599	KF-38789	−0.883	5.68E-07
3	BRD-K74305673	IKK-2-inhibitor-V	−0.675	3.46E-08
4	BRD-K01976263	emetine	−0.666	4.16E-10
5	BRD-K35960502	niclosamide	−0.605	4.16E-14
6	BRD-K64642496	KO-143	−0.587	3.45E-07
7	BRD-K94325918	kinetin-riboside	−0.573	6.22E-08
8	BRD-K77987382	mebendazole	−0.568	1.31E-09
9	BRD-K26997899	D-64131	−0.544	2.06E-07
10	BRD-K83289131	CAY-10618	−0.528	1.56E-07
11	BRD-K05653692	DL-PDMP	−0.459	1.62E-08
12	BRD-K33272502	DG-041	−0.454	5.52E-07
13	BRD-A19633847	perhexiline	−0.453	4.42E-07
14	BRD-K74514084	pazopanib	−0.417	3.31E-07
15	BRD-K97764662	PD-173074	−0.370	1.78E-07
16	BRD-A84481105	thioridazine	−0.326	5.04E-10

### Niclosamide Suppressed Calcium Deposition, Decreased *CROT* mRNA and Tissue Nonspecific Alkaline Phosphatase Activity in hSMCs Cultured in Osteogenic Condition

To validate the inhibitory effect of niclosamide on vascular calcification *in vitro*, we performed *CROT* mRNA expression analysis, and tissue nonspecific alkaline phosphatase (TNAP) activity and calcium deposition assays. We examined the effects of four concentrations of niclosamide (0.01, 0.03, 0.10, 0.30 μM) at 6, 24, and 72 h in cell culture and observed a dose-dependent decrease of *CROT* mRNA levels at 72 h in OM (*P* < 0.05, Figure 1C). Niclosamide at 0.3 μM increased mRNA level transiently at 6 h, but then reduced *CROT* mRNA levels dose dependently without cytotoxicity (Supplementary Figure IIB). OM induces TNAP, a key regulator of hSMC calcification (15). TNAP activity decreased after 7 days of niclosamide treatment in a dose-dependent manner (0.03-0.3 μM; *P* < 0.01) (Figure 1D). Alizarin red staining quantification showed that niclosamide decreased calcium deposition dose-dependently at 0.01 μM concentration (*P* < 0.001) (Figure 1E).

### Niclosamide Suppressed Vascular Calcification Without Decreasing Plasma Lipid Levels in High-Fat Diet-Fed *Ldlr*-Deficient Mice

**Figure 2:**
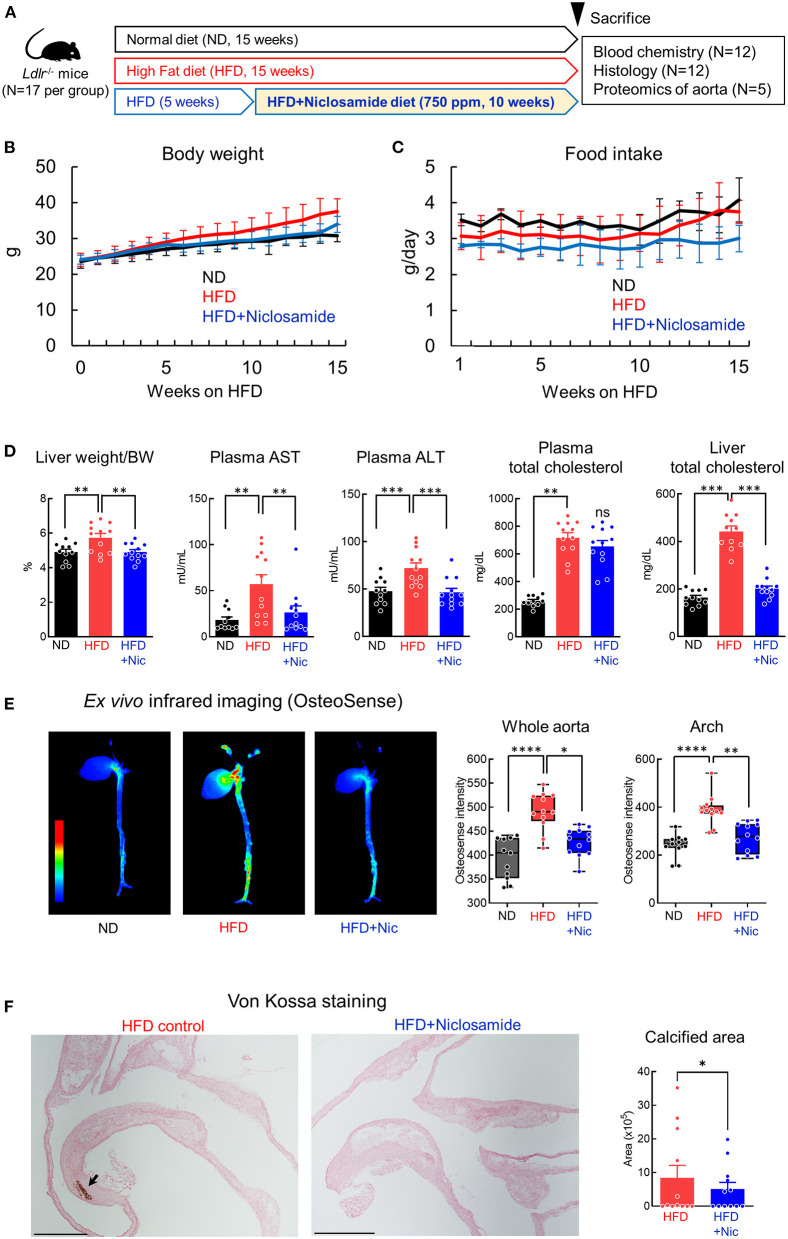
Niclosamide decreases cardiovascular calcification in *Ldlr*^−/−^ mice fed a high fat diet (HFD). **(A)** Schematic of the *in vivo* validation study using *Ldlr*^−/−^ mice treated with niclosamide (*N* = 12 mice per treatment group). **(B)** Body weight (BW) of mice fed normal diet (ND), HFD and HFD containing niclosamide from week 0 to 15. *N* = 12 mice per group, error bars represent mean ± SD. **(C)** Daily food intake for mice treated as in **(A)** from week 1 to 15. Each cage (*n* = 3) contained four animals per group; the food intake value per animal was calculated as average per each cage. Error bars represent mean ± SD. **(D)** Liver weight normalized to BW, plasma aspartate aminotransferase (AST) activity, plasma alanine aminotransferase (ALT) activity, plasma total cholesterol amount, and liver total cholesterol amount for mice treated as in **(A)** at terminal sacrifice. *N* = 12 mice per group, error bars represent mean ± SD, analyzed by ANOVA followed by Dunnett's multiple comparison test, **P < 0.01, ***P < 0.001, ****P < 0.0001. **(E)** *Ex vivo* near infrared fluorescence imaging and quantification using OsteoSense680 in mouse heart and aorta at terminal sacrifice. High intensity OsteoSense680 signal is shown in red, while low signal is in blue. *Box plots*—the accumulated signal intensity normalized by area calculated in a whole image and arch region; *N* = 12 mice per group, error bars represent mean ± SD, analyzed by ANOVA followed by Dunnett's multiple comparison test, *P < 0.05, **P < 0.01. **(F)** Representative von Kossa staining images of the aortic arch region for mice at terminal sacrifice. Calcified regions stain brown (arrow); scale bars = 2 mm. Calcified area quantification shown in graph, *N* = 12 mice per group, analyzed by Student *t*-test.

We used *Ldlr*^−/−^ mice to evaluate the effect of niclosamide on vascular calcification. *Ldlr* deficiency have previously demonstrated the formation of calcification in the mouse aorta (16). *Ldlr*^−/−^ mice received a normal diet (ND) or high-cholesterol/high-fat diet (HFD) for 15 weeks. For the niclosamide treatment group, the compound was admixed into HFD at 750 ppm concentration after 5 weeks feeding with HFD (Figure 2A). Body weight and food intake showed similar values among all groups (Figures 2B,C); however, when terminal body weight was considered alone, the HFD diet group's weight was higher (Supplementary Figure IIIA). HFD feeding induces steatosis; therefore, liver weight and plasma AST and ALT liver enzyme levels associated with liver damage were assessed. HFD increased liver weight, but niclosamide treatment mitigated this weight gain to the ND group's levels (*P* < 0.01, Figure 2D). Similarly, the HFD plus niclosamide treatment group's plasma AST and ALT levels were similar to those of the ND group, suggesting there is no apparent damage to the liver with niclosamide treatment (*P* < 0.001 or 0.01). Total cholesterol in the plasma and liver increased with HFD. Niclosamide reversed total cholesterol levels in the liver but did not change its plasma levels (*P* < 0.001, Figure 2D). The HFD group also showed increased plasma creatinine, but the HFD plus niclosamide group showed no change (Supplementary Figure IIIA). We further measured niclosamide plasma concentration by mass spectrometry. Our measurements showed that animals treated with 750 ppm niclosamide have 38.4 ± 19.8 ng/ml drug concentration in their plasma (Supplementary Figure IIIB). This concentration is converted to approximately 0.11 μM, which was at the same range as effective concentration in our *in vitro* study.

In addition, liver oil red O staining showed that niclosamide decreased lipid content compared to HFD control animals (Supplementary Figure IIID), suggesting lipid accumulation in the liver was averted. *Ex vivo* fluorescent reflectance near infrared molecular imaging using OsteoSense680 visualized higher signal of calcification in the aortic arch and carotid arteries in the HFD group. Niclosamide decreased calcification in the whole aorta and carotid arteries as compared to HFD alone (*P* < 0.05 or 0.01, Figure 2E). Von Kossa staining further demonstrated that niclosamide reduced calcification within aortic arch and carotid arteries (Figure 2F).

### Niclosamide Alters Wnt Signaling and RUNX Family Transcription Factor 2 Regulation

**Figure 3:**
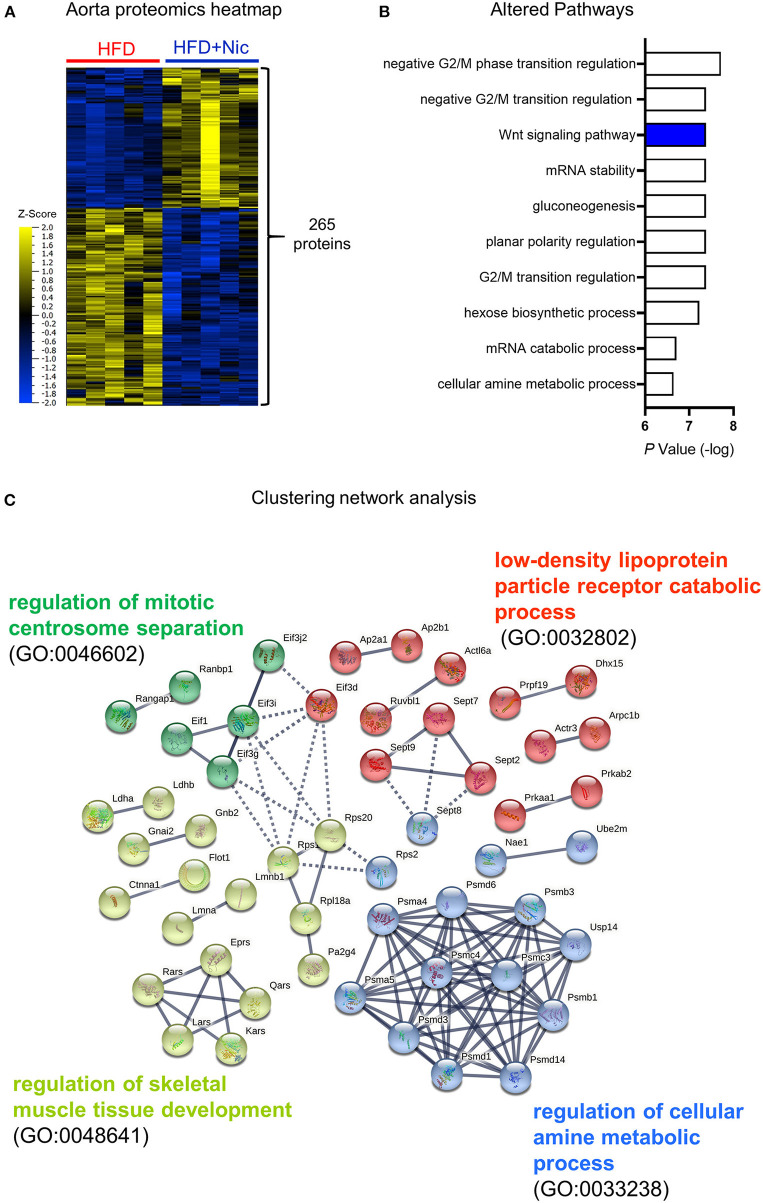
Proteomics and network analysis of aortas of mice treated with Niclosamide. **(A)** Hierarchical clustering and heat map of proteins that are differentially abundant in the aorta of mice fed a HFD vs. HFD containing niclosamide (a two-group comparison with *N* = 5 mice per group at terminal sacrifice, analyzed by F-*t-*test, *P* < 0.01). **(B)** Top ranked niclosamide altered pathways using gene ontology resources (GO biology process 2021). **(C)** Protein-protein (STRING database; showing physical subnetwork) and pathway analysis (Reactome database) including all identified 265 proteins in **(A)**. Most of Protein nodes in GO:0033238 involve Runx2 regulation and Wnt signaling pathway.

To investigate how niclosamide modifies biological processes in the aorta, we performed a proteomic analysis of aortic tissues from the ND, HFD and HFD plus niclosamide groups of *Ldlr*^−/−^ mice. Combined, the three treatment groups resulted in 1,939 proteins (with 2 or more unique peptides). We first performed a multigroup comparison to evaluate global changes across the three treatment groups; and noted that the two HFD groups (with or without niclosamide) were distinct from the ND group (Supplementary Figure IV). To determine proteins whose abundances changed specifically with niclosamide treatment, we performed a 2-group comparison the HFD and HFD plus niclosamide groups, yielding 265 differentially abundant proteins (Figure 3A). Pathway analysis showed that the Wnt signaling pathway was involved, as were pathways involving cell cycle regulation and energy/amino acid metabolism (Figure 3B). Protein-protein interaction combined with pathway analysis yielded a cluster of proteasome proteins that have been implicated in Wnt signaling and regulation of Runx2 expression (Figure 3C, blue cluster: Psma4, Psma5, Psmb1, Psmb3, Psmc3, Psmc4, Psmd1, Psmd3, Psmd6, and Psmd14) (17). A previous study reported that Runx2 is a crucial regulator of calcification through Wnt pathway (18).

### Niclosamide Acts Through Decreasing Runx2 Expression

**Figure 4:**
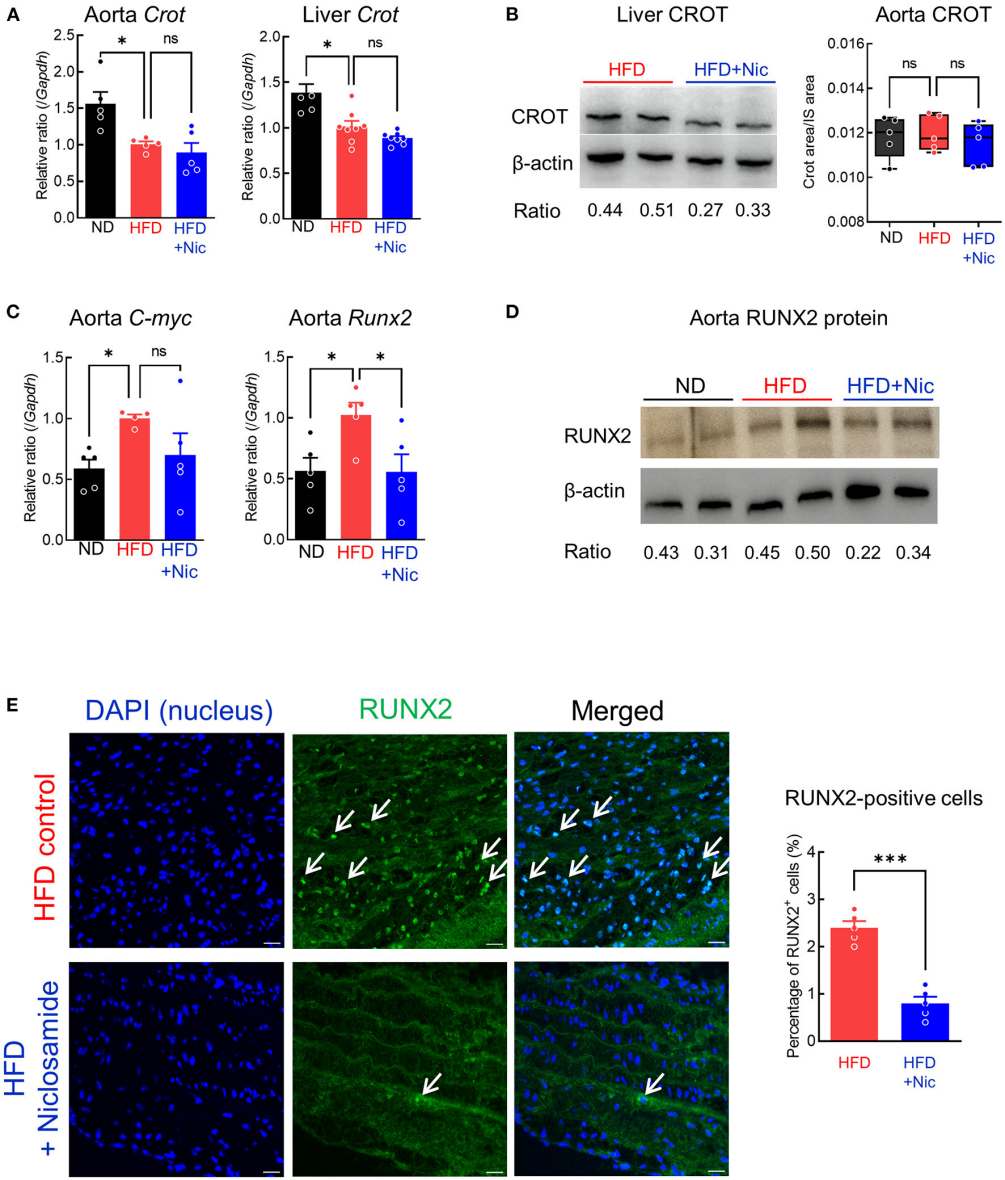
Niclosamide reduces Wnt signaling and Runx2 expression in the mouse aorta. **(A)** *Crot* transcript levels in the aorta and liver of mice treated with ND, HFD for 15 weeks or HFD containing niclosamide for 10 weeks. *N* = 5 (for the aorta) or 8 (for the liver) mice per group, error bars are mean ± SD, analyzed by ANOVA followed by Dunnett's multiple comparison test, *P < 0.05. **(B)** CROT protein levels in liver (Western blot) and the aorta (targeted mass spectrometry) of mice treated with ND, HFD and HFD containing niclosamide. Western blotting with anti-CROT antibody with liver sample (left, *N* = 2) from mouse treated HFD and HFD containing niclosamide. Average signal intensity ratio of CROT/β-actin are shown. CROT-targeted mass spectrometry peak area ratio in the aorta tissue (right), *N* = 5 mice per group analyzed by ANOVA-Dunnett's multiple comparison test, ns indicates not significant. **(C)** *c-myc* and *Runx2* transcript levels in the aorta of mice treated as in **(A)**. *N* = 5 mice per group, error bars represent mean ± SD, analyzed by ANOVA with Dunnett's multiple comparison test, *P < 0.05. **(D)** Western blotting with RUNX2 antibody with aorta samples from mice treated as in **(A)** (*N* = 2). Average signal intensity ratio of RUNX2/β-actin are shown. **(E)** Representative immunofluorescence images using anti-RUNX2 antibody for aorta tissue from mice treated as in **(A)**. Nuclei RUNX2 expression are shown (white arrows). Scale bars = 20 μm. RUNX2 positive cell number quantification shown in graph, *N* = 5 mice per group, analyzed by Student *t*-test. ***P < 0.001.

To support the notion that niclosamide decreases *Crot* mRNA expression in mice, we analyzed its transcriptional levels in the aorta and liver. In both tissues, the HFD group showed decreased levels of *Crot* mRNA expression as compared to ND (*P* < 0.05), but niclosamide did not induce further changes (Figure 4A). Using Western blotting, we demonstrated that liver CROT protein decreased with niclosamide treatment as compared to the HFD control (Figure 4B). Although *Crot* mRNA was detected in aorta, we did not detect its protein using this antibody. Therefore, we turned to targeted mass spectrometry to measure CROT, and in fact could detect and determine no change in protein abundance among three diet groups (Figure 4B). These results suggest that niclosamide may have inhibited calcification independent of *Crot* expression reduction. Since proteomics identified Wnt signaling as being altered in response to niclosamide, we examined the possibility that niclosamide is acting through the Wnt pathway.

To explore the Wnt pathway's potential contribution to the decrease in aortic calcification, we examined expression levels of the Wnt pathway target gene *C-myc*. While HFD *C-myc* mRNA levels in the aorta were higher than the ND, its level in the HFD plus niclosamide group were similar to those in the ND group (*P* < 0.05, Figure 4C). *Runx2* mRNA expression was also higher in the HFD group as compared to ND, but niclosamide decreased it to the levels of the ND group (*P* < 0.05, Figure 4C). RUNX2 protein levels were also higher in the HFD group than those in the ND and HFD plus niclosamide groups in the aorta, as determined by Western blotting (Figure 4D). Furthermore, fewer cells in the aorta of the HFD plus niclosamide group had RUNX2 protein in the nuclei as compared to HFD control (immunofluorescence, Figure 4E).

### Niclosamide Treatment Did Not Change Bone Density in *Ldlr*-Deficient Mice

**Figure 5:**
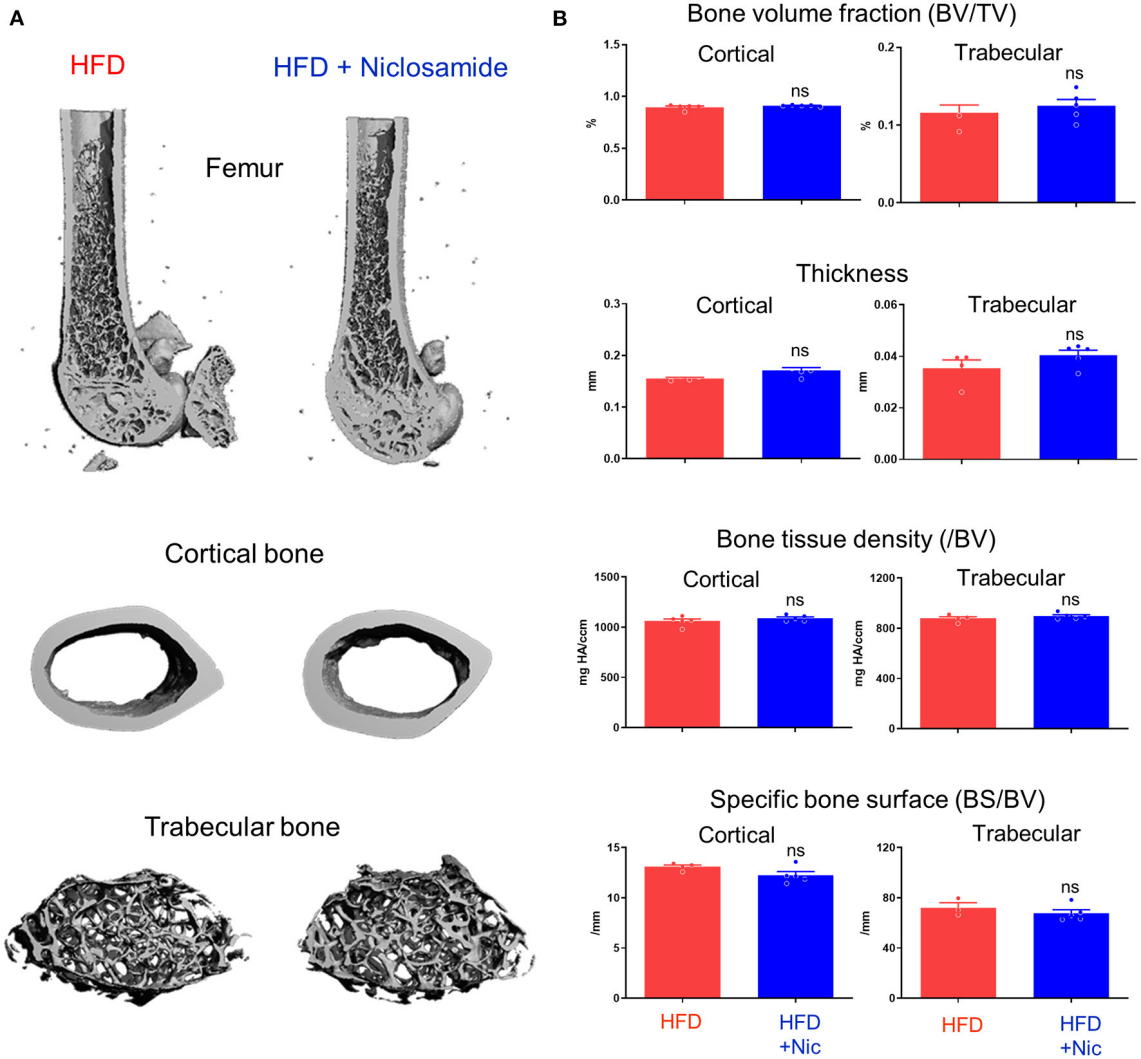
Niclosamide does not alter bone density in *Ldlr*^−/−^ mice fed with HFD. **(A)** Representative three-dimensional images of femur, cortical and trabecular bones from mice fed with HFD or HFD containing niclosamide. **(B)** Cortical and trabecular bone parameters measured by micro-CT. BV/TV, percentage bone volume (BV) in total volume (TV). Bone tissue density normalized by bone volume (/BV). Specific bone surface (BS) normalized by BV. *N* = 5 mice per group, error bars represent mean ± SD, analyzed by the Student's *t*-test, ns indicates not significant.

To examine the impact of niclosamide on bone density, we performed micro-computed tomography scanning of femur bones of *Ldlr*^−/−^ mice fed a HFD and treated with niclosamide. Mass and structure of cortical shaft of femur bones showed no difference between groups (Figure 5A). Niclosamide treatment did not change femur cortical or trabecular bone volume, thickness, tissue density or specific bone surface (Figure 5B). These results demonstrate that niclosamide can suppress vascular calcification without affecting bone density in atherosclerotic mice, a desirable property of cardiovascular anti-calcification drug.

## Discussion

In the present study, we employed a computational approach to screen small molecules that could decrease the expression of *CROT*, a novel target for inhibition of vascular calcification previously reported by our group (4). We then assessed their ability to decrease *CROT* mRNA and calcification potential *in vitro* and *in vivo*, resulting in the identification of niclosamide, an FDA approved anthelmintic drug that has been widely used for the treatment of tapeworm infestations in humans (11, 12). This *in silico* screen initially identified 13 candidate compounds from a pool of more than 10,000 small molecules. Among these candidates only niclosamide substantially decreased *CROT* transcriptional levels and calcium deposition in hSMCs cultured in an osteogenic condition. On the other hand, niclosamide did not significantly decrease *Crot* transcriptional levels in the aorta of *Ldlr*^−/−^ mice fed with HFD while genetic abrogation of *Crot* in *Crot*^−/−^*Ldlr*^−/−^ mice significantly reduced calcification (4). However, the RT-PCR threshold cycle values indicated that *Crot* mRNA expression in aorta is considerably lower than in the liver. In our global proteomic analysis, we did not detect CROT protein in the aorta, but the more sensitive targeted mass spectrometry analysis demonstrated that CROT protein levels did not differ despite niclosamide's inhibition of vascular calcification. Our proteomic analysis of the mouse aorta pointed to a potential explanation for this *in vitro-in vivo* paradox. Pathway analysis revealed that niclosamide changed the expression profiles of proteins associated with Wnt signaling. Canonical Wnt pathway regulates long chain fatty acid metabolism through beta catenin (19). Our previous work reported that CROT contributes to vascular calcification via promoting fatty acid metabolism (4) thus linking Wnt and CROT pathways. Additional studies are needed to investigate the CROT-Wnt axis in cardiovascular calcification.

Previous studies demonstrated that niclosamide targets several signaling pathways including STAT3 (20, 21), mTORC1 (22), NF-κB and Notch (23, 24), in addition to Wnt (25, 26). Wnt, Notch or STAT3 regulates or inhibits vascular calcification (27–29). Wnt signaling comprises multiple pathways and regulates vascular calcification. In regard to osteogenesis, the Wnt pathway plays crucial role in regulation of bone formation by controlling osteoblast differentiation through Runx2 (30). In hSMCs, Wnt3a, a canonical Wnt pathway ligand, induced calcium deposition (31). In another study using phosphate and Bone Morphogenetic Protein 2 (BMP2) as inducers of calcification in hSMCs, osteogenic markers such as RUNX2 and beta-catenin were increased (32). β-catenin deletion reversed these results. Canonical Wnt pathway activation through the Wnt3a ligand induced RUNX2 expression to promote calcification in hSMCs (18). The present study demonstrated that niclosamide suppressed Wnt pathway and decreased Runx2 mRNA and protein expression in the atherosclerotic aorta of *Ldlr*^−/−^ mice. These findings support the notion that niclosamide inhibits vascular calcification through altering the Wnt pathway.

*Ldlr*^−/−^ mice fed a HFD develop hepatic steatosis with metabolic disfunction (33). In this study, we demonstrated that niclosamide treatment reduced steatosis almost to levels of the normal diet control; consistent with the results of another niclosamide study using diabetic hyperlipidemic mice (34). Since niclosamide did not change plasma total cholesterol levels, it may suggest that cholesterol liver intake from blood decreased. Our results are consistent with a report on the ethanolamine salt of niclosamide that improved cellular metabolism in the liver by increasing energy expenditure and lipid oxidation through AMP-activated protein kinase (AMPK) pathway activation (34). On the other hand, unchanged total plasma cholesterol by niclosamide treatment suggests that this compound inhibits calcification independently of lipid lowering. Plasma cytokines promote vascular calcification through chronic inflammation (35). In our study, chemerin levels in the plasma decreased in association with vascular calcification. Chemerin is adipokine associated with inflammation and lipid metabolism that attenuates vascular calcification in mice (36). Since *Rarres2*, encoding chemerin, did not change in the liver, niclosamide did not act through cytokines to mitigate calcification. The evidence has linked vascular calcification, especially in the coronary artery, and hepatic steatosis in patients (37). Further studies would investigate the interplay between pathological changes in the liver and vascular calcification.

*Ldlr*-deficient mice develop intimal calcification that is accompanied by high levels of inflammatory cytokines, hyperlipidemia, or metabolic syndrome (38). Vascular intimal calcification correlates with atherosclerotic plaque and microcalcification in fibrous plaque and promotes mechanical stress and plaque rupture (39). In this study we did not evaluate calcification in the aortic valve, which is a major risk factor for heart disease. In the future, the effects of niclosamide on valve calcification should be elucidated as the Wnt pathway can regulate valve calcification through phosphate metabolism or valve leaflet stratification (40, 41). Although its role in inhibiting vascular calcification is not yet fully clarified, the evidence suggests that niclosamide targets several signaling pathways including Wnt-β catenin. In addition, further studies will be required to establish safety profile when intending to use niclosamide or its derivatives for cardiovascular treatment. Niclosamide showed advantage for its safety, as shown in many reports suggesting repositioning of this drug due to its excellent safety profile as was demonstrated with decades of clinic use (42, 43).

In conclusion, we identified a small molecule, niclosamide, that inhibits calcification *in vivo* and *in vitro*. We suggest that niclosamide suppresses Runx2 through Wnt pathway inhibition in the aorta with no detrimental effects on liver or bone. Our computational approach using CMap and the L1000 database demonstrated that genetic perturbation screens can be useful to identify existing drugs. Compounds like niclosamide can be repositioned with novel indications, including repurposing for the treatment of vascular calcification.

## Data Availability Statement

The datasets presented in this study can be found in online repositories. The names of the repository/repositories and accession number(s) can be found at: ProteomeXchange, PXD029963.

## Ethics Statement

The animal study was reviewed and approved by Beth Israel Deaconess Medical Center's Institutional Animal Care and Use Committee.

## Author Contributions

TT: conception and design, collection of data, data analysis and interpretation, manuscript writing, and final approval of the manuscript. TA and SS: conception and design, collection of data, data analysis and interpretation, and final approval of the manuscript. TO: collection of data, data analysis and interpretation, and final approval of the manuscript. SK: data analysis and interpretation, manuscript writing, and final approval of the manuscript. MA: conception of the use of computational drug screening, financial support, administrative support, and final approval of the manuscript. EA: conception and design, financial support, administrative support, data interpretation, manuscript writing, and final approval of the manuscript. All authors contributed to the article and approved the submitted version.

## Funding

This study was supported by a research grant from Kowa Company, Ltd., (Tokyo, Japan, to MA) and the National Institutes of Health grants (R01HL136431, R01HL147095, and R01HL141917 to EA).

## Conflict of Interest

TT, TA, TO, and SK are employees of Kowa Company, Ltd., and were visiting scientist at Brigham and Women's Hospital when experiments included in this study were performed. This study received funding from Kowa Company, Ltd., (Japan). Kowa Company, Ltd., had no role in study design, data collection and analysis, decision to publish or preparation of the article. The remaining authors declare that the research was conducted in the absence of any commercial or financial relationships that could be construed as a potential conflict of interest.

## Publisher's Note

All claims expressed in this article are solely those of the authors and do not necessarily represent those of their affiliated organizations, or those of the publisher, the editors and the reviewers. Any product that may be evaluated in this article, or claim that may be made by its manufacturer, is not guaranteed or endorsed by the publisher.
