# Supplementary material for: Computational Screening Strategy for Drug Repurposing Identified Niclosamide as Inhibitor of Vascular Calcification
Source: Front Cardiovasc Med. 2022 Jan 20;8:826529. doi: 10.3389/fcvm.2021.826529 (PMC8811128; doi:10.3389/fcvm.2021.826529)
Supplement: Supplementary file 1 [file Data_Sheet_1.docx]

Supporting Information

Computational screening strategy for Drug Repurposing Identified Niclosamide as Inhibitor of Vascular Calcification

Takeshi Tanaka^1^, Takaharu Asano^1^, Takehito Okui^1^, Shiori Kuraoka^1^, Sasha A. Singh^1^, Masanori Aikawa^1,2,3^, Elena Aikawa^1, 2,3*^

^1^Center for Interdisciplinary Cardiovascular Sciences and ^2^Center for Excellence in Vascular Biology, Cardiovascular Medicine, Brigham and Women's Hospital, Harvard Medical School, Boston, MA 02115, USA; ^3^Department of Human Pathology, Sechenov First Moscow State Medical University, Moscow 119992, Russia.

*** Correspondence:**

Elena Aikawa, MD, PhD

Brigham and Women’s Hospital

3 Blackfan St, 17th Floor, Boston, MA 02115

Phone: 617-730-7755; Fax: 617-730-7791
Email: eaikawa@bwh.harvard.edu

Keywords: Calcification, drug discovery, drug repurposing, mouse models, proteomics, Wnt signaling.

# Supplemental methods

## Quantitative real-time PCR

RNA of human coronary artery smooth muscle cells (hSMCs) was collected using TRIzol® reagent (15596-018, Thermo Fisher Scientific Inc.) at 6, 24 or 72 hours after switching from NM to OM (or NM for controls) containing test compounds. RNA of mice aorta or liver were collected at terminal sacrifice. The tissues were homogenized with a Precellys 24 homogenizer and magnetic beads (Bertin Instruments). Total RNA was extracted using the manufacturer’s protocol. cDNA was synthesized from 1 μg of total RNA from each sample using qScript cDNA Synthesis Kit (95047, Quantabio) according to the manufacturer’s protocol. PCR reactions were performed using PerfeCTa® qPCR FastMix® II, ROX™ (Quantabio, Cat#: 95119), TaqMan gene expression assay primers are listed in the Resources Table. Real time PCR was performed on a 7900HT Fast Real-time PCR system or QuantStudio 5 Real-time PCR system (Thermo Fisher Scientific Inc.). The relative value of mRNA abundance was calculated with threshold cycle values that were first normalized to those of glyceraldehyde 3-phosphate dehydrogenase gene. The relative differences in gene expression compared were calculated using the 2^-ΔΔ­­C^*_T_* methods.

## TNAP activity assay

hSMCs were cultured in 24-well plates for 7 days in either NM or OM media, and then switched to fresh NM, OM, or OM supplemented with niclosamide (0, 0.03, 0.1 and 0.3 μM). TNAP enzymatic activity was measured using the Alkaline Phosphatase Activity Colorimetric Assay Kit (K413, BioVision Inc.) Protein lysates were collected with ALP assay buffer, sonicated and centrifuged 1,0000 *g* for 10 minutes at 4°C.

## Cell viability assay

SMCs were treated with niclosamide in OM for 72 hours in 96-well plates, and the assay was performed by using Cell Counting Kit 8 (WST-8/CCK8) (ab228554, Abcam).

## Alizarin red staining

hSMCs cultured (in 48-well plates) for 21 days in NM, OM, or OM supplemented with niclosamide (0, 0.01, 0.03, 0.1 and 0.3 μM) were stained with Alizarin red. Cells were washed with PBS twice, fixed with 4% formaldehyde for 10 minutes, then stained with 2% Alizarin red stain (CM-0058, Lifeline Cell Technology) for 15 minutes. Stained cells were washed three times with water, and images were taken. Stains were extracted with 10 mmol/L of cetylpyridinium chloride solution (40300072, bioPLUS fine research chemicals) for 30 minutes and the absorbance at 540 nm was measured with a microplate reader (SpectraMax i3x, Molecular Devices).

## Cholesterol, triglyceride, aspartate aminotransferase, and alanine aminotransferase assays with plasma and liver samples

Whole blood samples were drawn from the inferior vena cava with heparinized syringes and centrifuged at 3,000 *g* for 10 minutes at 4°C. Plasma was separated and frozen at -80°C. After perfusion from the left ventricle by 10 mL of PBS, the liver was removed and frozen in liquid nitrogen, then stored at -80°C. Liver samples were minced and homogenized using a Precellys 24 tissue homogenizer (Bertin Instruments) with CK14 ceramic beads. Protein amount was measured using the Pierce BCA Protein Assay Kit (23225, Thermo Fisher Scientific Inc.) for normalization. Aspartate aminotransferase (AST), alanine aminotransferase (ALT), triglyceride and total cholesterol levels in plasma, and total cholesterol in the liver were measured using commercial kits obtained from FUJIFILM Wako Diagnostics USA Corporation (cholesterol: Cat#: 439-17501, triglycerides: Cat#: 992- 02892 and 998-02992). or MilliporeSigma (AST: Cat# MAK055-1KT, ALT: Cat# MAK052-1KT) as manufacturer’s instruction.

## Immunofluorescence

Aortic tissue samples were embedded in Premium Frozen Section Compound (OCT; VWR International, LLC., Cat#: 95057-838) and frozen. Six μm fresh frozen sections were cut using a cryostat (Leica Biosystems) and fixed with 4% paraformaldehyde for 5 minutes. After rinsing with water twice and washing twice with PBS for 5 minutes, sections were incubated with 0.3% hydrogen peroxide for 3 minutes. Sections were rinsed with water and then with PBS for 5 minutes. Sections were covered with 4% normal serum for 30 minutes for blocking. After tapping off blocking serum, primary antibody solution was applied on the sections and incubated for 1 hour. Sections were washed twice with PBS for 5 minutes and incubated with secondary antibodies for 30 minutes. After washing twice with PBS for 5 minutes, sections were then incubated with DAPI (4',6-Diamidino-2-Phenylindole, Dihydrochloride) (Thermo Fisher Scientific Inc., Cat#: D1306) for nuclear counterstaining. Imaging and analysis were performed using confocal microscopy on a Nikon confocal A1 scope (Nikon Corporation).

## Histological analysis of mouse aorta and liver

Unfixed tissue samples were frozen in OCT compound and serial sections were routinely prepared. Von Kossa silver stain was used to visualize inorganic phosphate calcium deposition. The sections were incubated with 5% silver nitrate (American Master Tech Scientific) for 60 minutes under UV light, then washed with sodium thiosulfate. Nuclei were stained with nuclear fast red (American Master Tech Scientific). Then, we traced and quantified plaque and calcification area using NIS Elements 3.10 software (Nikon). Oil red O staining was used to visualize lipid contents in the liver. The liver sections were fixed in formalin and stained with Oil Red O for 15 minutes. Nuclei were stained with hematoxylin.

## Micro-computed tomography (CT) bone density scanning

Femur bone samples were collected at terminal sacrifice after perfusion and stored in 70% ethanol at 4°C. Scanning was performed at the Yale Core Center for Musculoskeletal Disorders micro-CT Facility. Femurs were analyzed in 70% ethanol by cone beam microfocus x-ray computed tomography using a Scanco μCT-35 instrument (Scanco, Brutissellen, Switzerland). Images were acquired at 55 kVp, with an integration time of 500 ms and an isometric voxel size of 6 µm. Segmentation of bone from marrow and soft tissue was performed in conjunction with a constrained Gaussian filter (support=1; 3×3×3 voxel window; σ=0.8) to reduce noise, applying density thresholds of 250 and 420 for trabecular and cortical compartments of femur, respectively. Volumetric regions for trabecular analysis were selected within endosteal borders of distal femoral metaphysis to include secondary spongiosa located 1 mm from growth plate and extending 1 mm proximally, or from within cortical shell of the third lumbar vertebral body. Cortical morphometry was quantified and averaged volumetrically through 233 serial cross-sections (1.4 mm) centered on the diaphyseal midpoint between proximal and distal growth plates.

## Niclosamide plasma concentration measurement

For quantification of plasma niclosamide concentrations, niclosamide was extracted from 20 μL plasma samples by adding 20 μL of methanol and 200 μL of the oxyclozanide solution (34078, Sigma Aldrich Co., 50 ng/mL in methanol, used as an internal standard). In addition, 20 μL of a niclosamide standards (N3510, Sigma Aldrich Co., 0.3-100 ng/mL in methanol) were mixed with 20 μL of mouse plasma (devoid of niclosamide), and 200 μL of the oxyclozanide solution. Samples were centrifuged at 10,000 g for three minutes at 4°C. Supernatants were diluted by 0.1% formic acid (1:1 v/v) and used for mass spectrometry analysis.

## Mass spectrometry

Mouse aortic tryptic peptides (total 15 peptide samples, three mouse groups) were analyzed by data-dependent mass spectrometry (global proteomics) and by targeted CROT mass spectrometry, using an Orbitrap Fusion Lumos Tribrid mass spectrometer (Thermo Fisher Scientific) fronted with an Easy-Spray ion source, and coupled to an Easy-nLC1000 HPLC pump (Thermo Fisher Scientific). Peptides were separated using a dual column set-up: an Acclaim PepMap 100 C18 trap column, 75 µm X 20 mm; and an EASY-Spray HPLC heated (45 oC) column, 75 µm X 250 mm (Thermo Fisher Scientific). For global proteomics, the gradient flow rate was 300 nL/min from 5 to 21% solvent B (acetonitrile/0.1% formic acid) for 50 minutes, 21 to 30 % solvent B for 10 minutes, followed by 95% solvent B for 10 minutes. The instrument was set to 120 K resolution, and the top N precursor ions in a 3 second cycle time (within a scan range of m/z 375-1500; isolation window, m/z 1.6; ion trap scan rate, normal) were subjected to collision induced dissociation (CID; collision energy, 30%) for peptide sequencing (or MS/MS). Dynamic exclusion was enabled (30 seconds). For CROT targeted mass spectrometry, CROT peptides (ILNDVSQAK, a.a. 375-383; SGNTPLDMNQFR, a.a. 148-159), YWHAB peptides (YLSEVASGENK, a.a. 129-139; YLILNATQAESK, a.a. 105-116) and VDAC1 peptides (VTQSNFAVGYK, a.a. 176-186; LTFDSSFSPNTGK, a.a. 109-121) were measured. The analytical gradient was run at 300 nL/min from 5 to 21% Solvent B for 20 minutes, 21 to 35% Solvent B for five minutes, followed by the jigsaw wash. Precursor m/z values were 494.2771 for ILNDVSQAK (charge, 2; time window 7.0-8.5 min), 698.3197 for SGNTPLDMNQFR (M8-oxidized; charge, 2; time window 11.5-13.5 min), 598.7933 for YLSEVASGENK (charge, 2; time window 8.0-10.0 min), 675.8675 for YLILNATQAESK (charge, 2; time window 15.0-17.0 min), 607.3143 for VTQSNFAVGYK (charge, 2; time window 11.0-13.0 min), 700.8387 for LTFDSSFSPNTGK (charge 2; time window 16.0-18.5 min) using an isolation window of 1.2 m/z. The MS/MS spectra (HCD; collision energy, 30%; scan range, m/z 350-1200) were scanned at 60K resolution (Orbitrap). Plasma samples were analyzed by targeted mass spectrometry using a Q Exactive coupled to a Vanquish UHPLC systems (Thermo Fisher Scientific). Niclosamide and oxyclozanide were separated using an Accucore Vanquish C18+ UHPLC column (100 mm X 2.1 mm, 1.5 μm, Thermo Scientific) heated at 55 °C. The gradient flow rate was 0.450 mL/min from 5% to 95% solvent B (0.1% formic acid in methanol) for four minutes, 95% solvent B for two minutes, followed by the jigsaw wash. Solvent A was 0.1% formic acid in water. The mass spectrometer was operated in negative ion mode. Precursor m/z values were 324.98 for Niclosamide (retention time = 4.4 min) and 397.87 for oxyclozanide (4.1 min) using an isolation window of 5 m/z. The MS/MS spectra (HCD; collision energy, 30%) were scanned at 70 K resolution (Orbitrap).

## Mass spectrometry data analysis

For global proteomics, the 15 mass spectral files were queried against the mouse UniProt database (n=63,703 entries; downloaded September 09, 2020) using the SEQUEST-HT search algorithm, via the Proteome Discoverer (PD) Package (version 2.2, Thermo Fisher Scientific). Trypsin (full) was set as the digestion enzyme, allowing up to 4 missed cleavages and a minimum peptide length of 4 amino acids. A 10 ppm tolerance window was used in the MS1 search space, and a 0.6 Da fragment tolerance window was used for CID. Methionine oxidation and n-terminal acetylation were set as dynamic modifications, and carbamidomethylation of cysteine was set as a static modification. Peptides were filtered based on a 1% FDR based on the reverse database results. In order to quantify peptide precursors detected in the MS1 but not sequenced from sample to sample, we enabled the ‘Feature Mapper’ node to quantify proteins across 15 datasets. Chromatographic alignment was done with a maximum retention time (RT) shift of 10 minutes and a mass tolerance of 10 ppm. Feature linking and mapping settings were, RT tolerance minimum of 0 minutes, mass tolerance of 10 ppm and signal-to-noise minimum of five. Precursor peptide abundances were based on their chromatographic intensities and total peptide amount was used for normalization. Unique and razor peptides were used for quantification. Peptides assigned to a given protein group, and not present in any other protein group, were considered as unique. Consequently, each protein group is represented by a single master protein (PD Grouping feature). The final protein list was filtered for 2 or more unique peptides. Targeted CROT data were quantified with Skyline (version 21.1.0.146, Maclean et al., 2010). Peak area ratio (total area of CROT fragments/total area of YWHAB and VDAC1 fragments) was calculated using the following fragments: ILNDVSQAK (CROT), y5 m/z 532.31, y6 m/z 647.34, y5 m/z 532.31; SGNTPLDMNQFR (CROT, M8-oxidized), y5 m/z 711.32, y6 m/z 826.35, y7 m/z 939.44; YLSEVASGENK (YWHAB), y6 m/z 605.29, y7 m/z 704.36, y8 m/z 833.40; YLILNATQAESK (YWHAB), y7 m/z 734.37, y8 m/z 848.41, y9 m/z 961.49; VTQSNFAVGYK (VDAC1), y6 m/z 684.37, y7 m/z 798.41, y8 m/z 885.45; VTQSNFAVGYK (VDAC1), y7 m/z 750.38, y8 m/z 837.41, y9 m/z 924.44. Targeted niclosamide data were quantified with Skyline (version 21.1.0.146). Peak area ratio (niclosamide/oxyclozanide) was calculated using the following ions: niclosamide, m/z 324.98 and 289.00; oxyclozanide, m/z 201.95. A standard curve (weighting factor, 1/w) was constructed by plotting peak area ratio (y) vs nominal concentration of niclosamide (x) and used for quantification of plasma niclosamide concentration.

## Mouse aorta proteomic data analysis

We used the Qlucore Omics Explorer (version 3.7 Qlucore, Sweden) to identify proteins whose abundances were altered by niclosamide treatment. 265 differentially abundant proteins were identified when the HFD control and niclosamide treatment groups were compared (F-*t* test, p<0.01). STRING (ELIXIR infrastructure, Szklarczyk et al., 2021) was used to generate a protein-protein interaction network (using 0.900 as an interaction score cut-off); and k-means clustering was used to identify candidate input proteins for network analysis using EnrichR (Chen et al., 2013). We queried all 265 proteins or individual clusters into EnrichR to identify altered biological pathways using the gene ontology resource (GO biological process 2021).

**
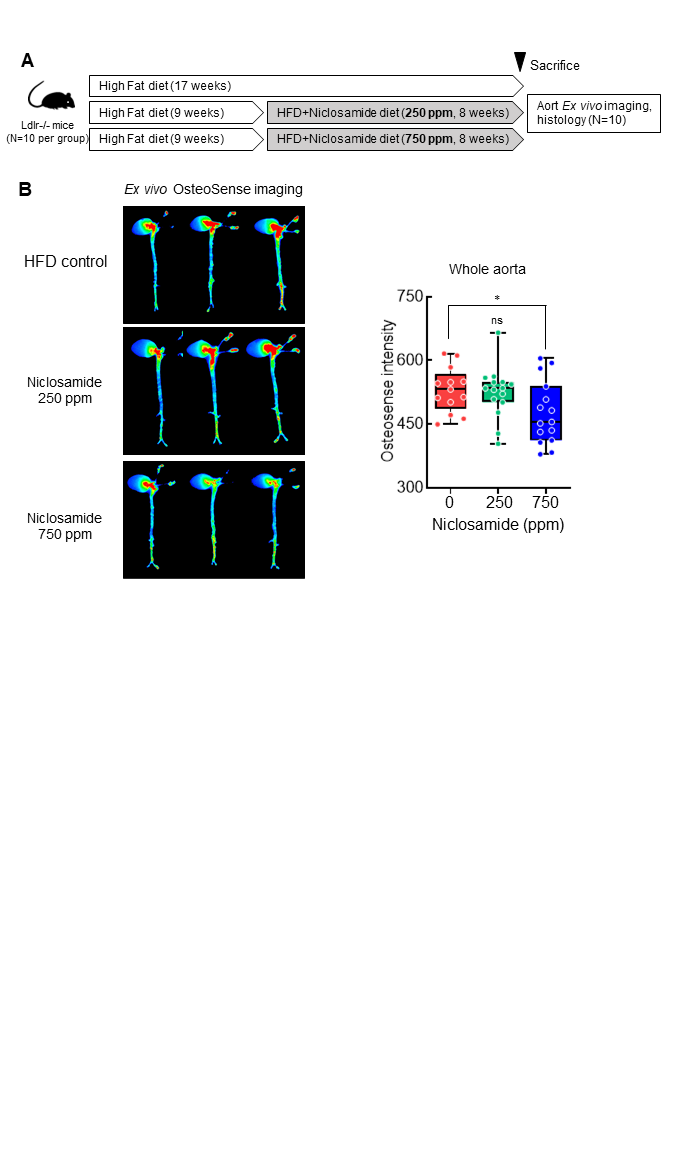
Supplemental Figure I. Pilot study using *Ldlr^-/-^* mice treated with a high fat diet (HFD) containing niclosamide for 8 weeks.**

**A.** Cartoon outline of the pilot study using *Ldlr^-/-^* mice treated with niclosamide in two doses. **B.** *Ex vivo* near-infrared fluorescence imaging and quantification using OsteoSense680 in mouse heart and aorta at terminal sacrifice. High intensity of OsteoSense680 shown in red while low in blue. Accumulated signal intensity normalized by area calculated in whole image. N=10 mice per group, error bars are mean±SD, analyzed by ANOVA with Dunnett’s multiple comparison test, **P*<0.05.

**
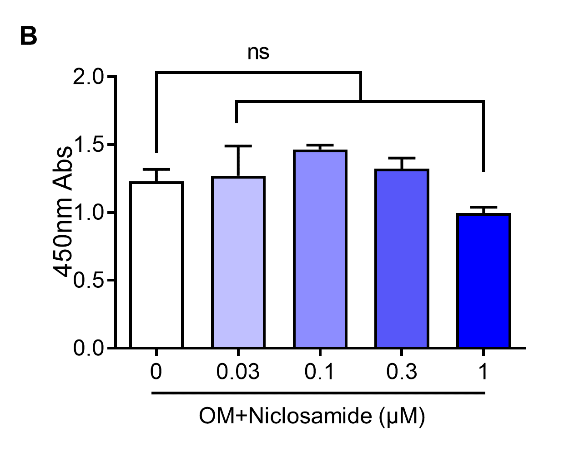

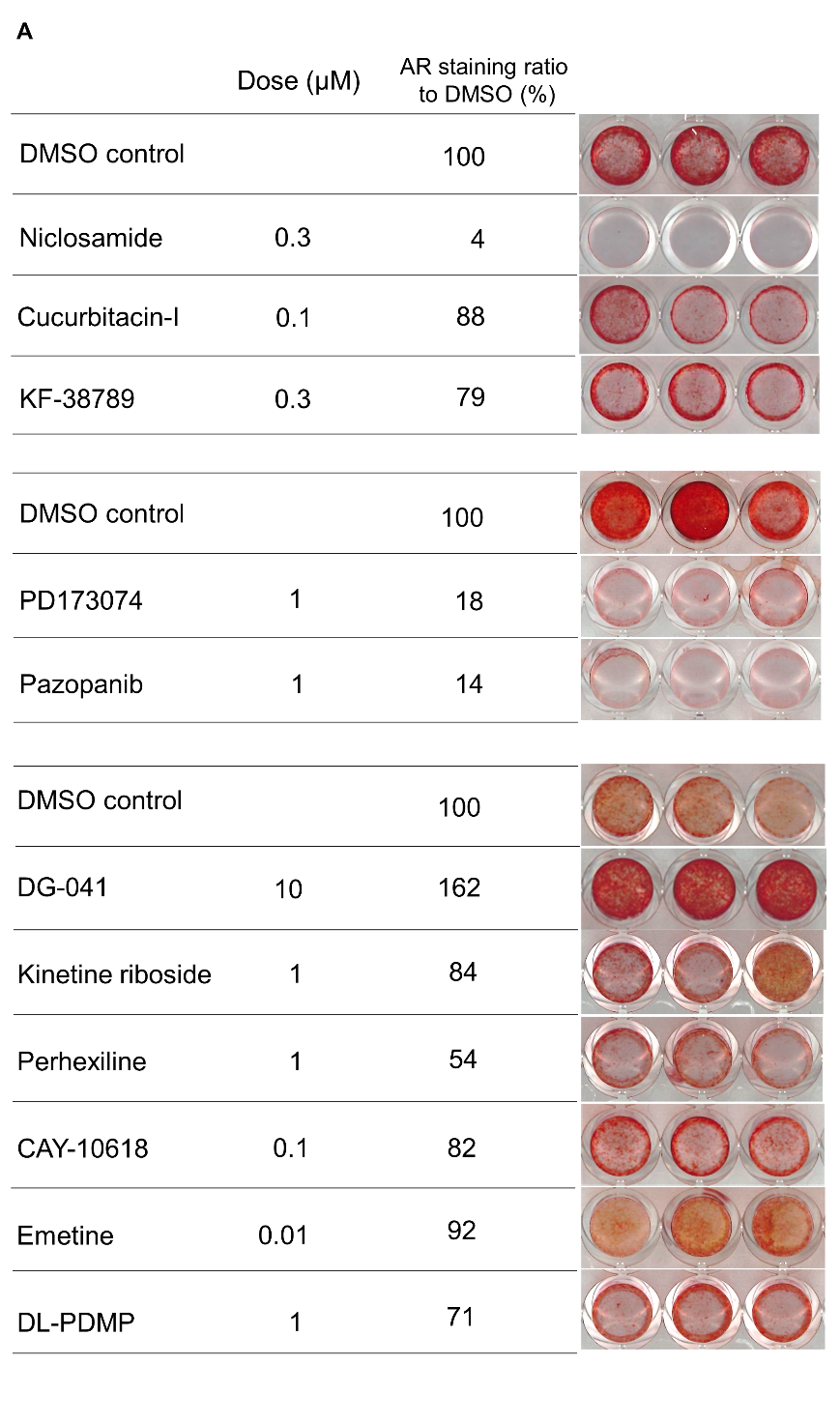
**

**Supplemental Figure II. Niclosamide strongly suppressed calcium deposition in compound screening study using human smooth muscle cells (hSMCs).**

**A**. Alizarin red (AR) staining and quantification in hSMCs treated with several different compounds for 21 days. Niclosamide showed strongest inhibition. Calcium deposition assay was performed using a single donor, prepared in three compound batches (with a DMSO control for each batch); each compound had 3 replicates. AR staining quantification is shown as a ratio to each DMSO control. **B**. Cytotoxicity assay in hSMCs treated with niclosamide for 72 hours. 6 replicates, error bars are mean±SD, analyzed by ANOVA with Dunnett’s multiple comparison test, ns indicates not significant.


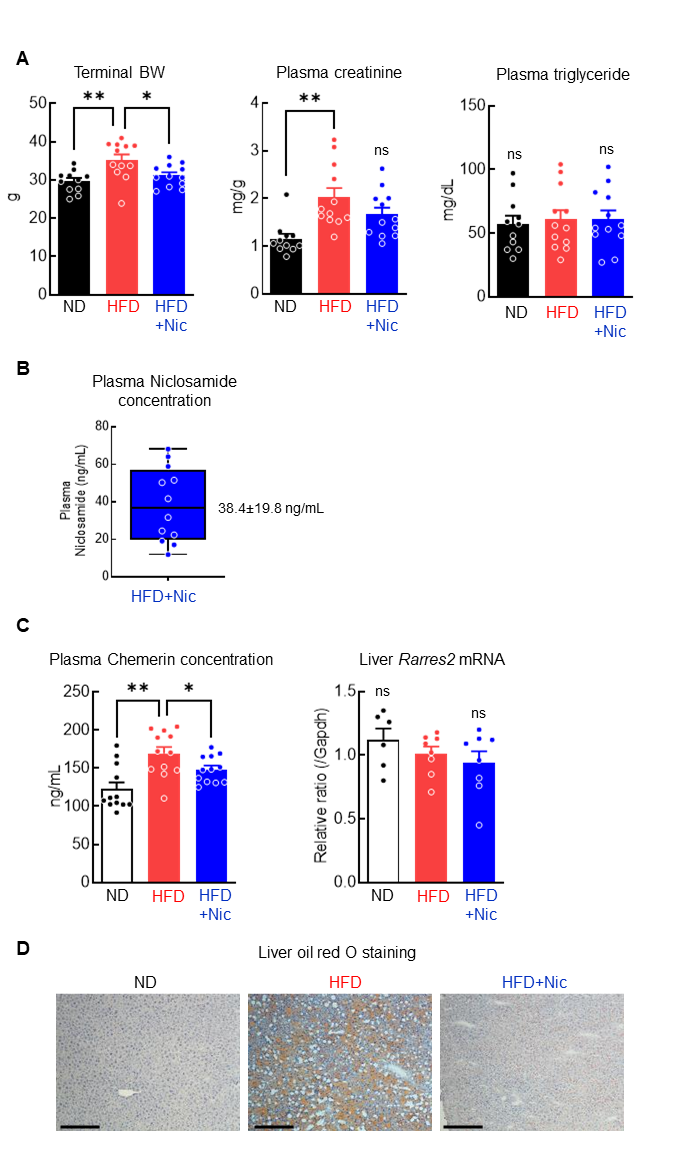


**Supplemental Figure III. Blood chemistry and plasma concentration of niclosamide in *Ldlr^-/-^* mice treated with high fat diet (HFD) containing niclosamide.**

**A.** Terminal body weight, plasma creatinine and plasma triglyceride for mice with normal diet (ND), HFD, and HFD containing niclosamide (750 ppm). **B**. Plasma niclosamide concentration for mice received HFD containing 750 ppm niclosamide for 10 weeks. **C**. Plasma chemerin concentration for mice treated as in **A**. **D**. Representative oil red O staining images in the liver for mice treated as in **A**. Lipid contents shown depicted with red brown color. Scale bars represent 2 mm. (**A-C**) N=12 mice per group, error bars are mean±SD, analyzed by ANOVA with Dunnett’s multiple comparison test, **P*<0.05, ***P*<0.01, ns indicates not significant.

**
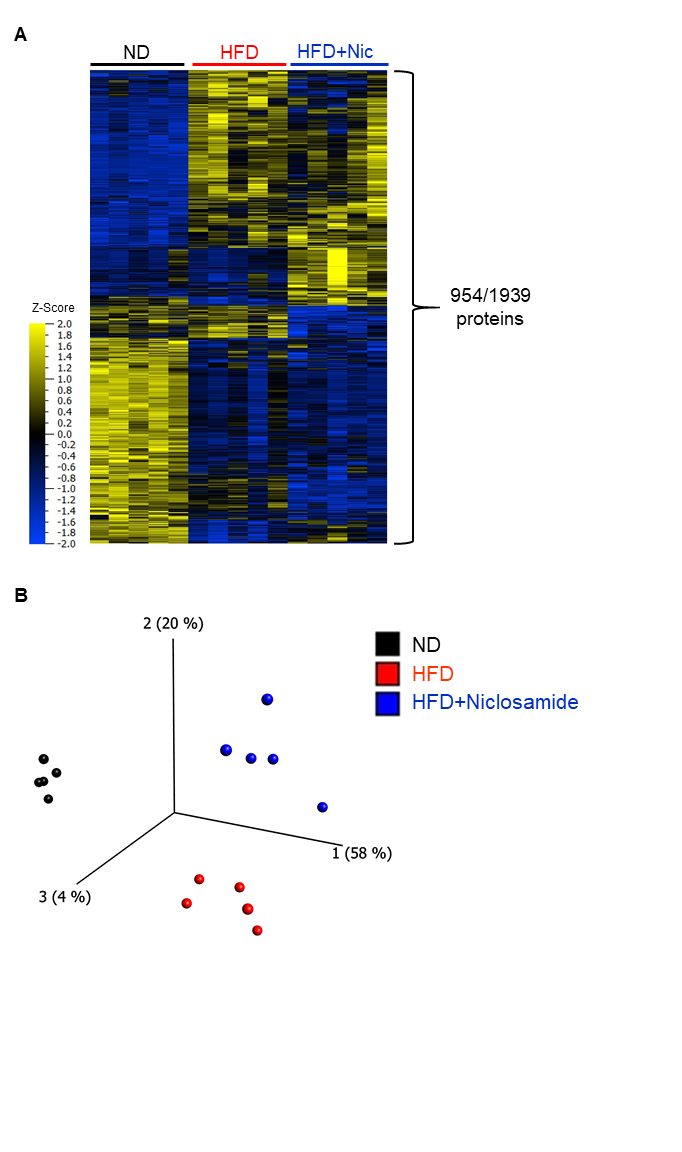
Supplemental Figure IV. Proteomics heat map of differential protein expression changes in the aorta.**

**A.** Hierarchical analysis and heat map of proteins after a multigroup comparison across normal diet (ND), high fat diet (HFD) and HFD plus niclosamide. N=5 mice per group at terminal sacrifice, analyzed by Dunnett test, P<0.01. **B**. PCA plot of proteins that analyzed as in **A**.

**References**

Chen EY, Tan CM, Kou Y, et al. Enrichr: interactive and collaborative HTML5 gene list enrichment analysis tool. BMC Bioinformatics. 2013;14:128. Published 2013 Apr 15. doi:10.1186/1471-2105-14-128

MacLean B, Tomazela DM, Shulman N, Chambers M, Finney GL, Frewen B, Kern R, Tabb DL, Liebler DC, MacCoss MJ. Skyline: An Open Source Document Editor for Creating and Analyzing Targeted Proteomics Experiments. Bioinformatics. 2010;26(7):966-968. doi: 10.1093/bioinformatics/btq054.

Szklarczyk D, Gable AL, Nastou KC, et al. The STRING database in 2021: customizable protein-protein networks, and functional characterization of user-uploaded gene/measurement sets [published correction appears in Nucleic Acids Res. 2021 Oct 11;49(18):10800]. Nucleic Acids Res. 2021;49(D1):D605-D612. doi:10.1093/nar/gkaa1074

**Resources Table**

**Antibodies**

| Target antigen | Vendor | Catalog # | Working concentration |
| --- | --- | --- | --- |
| Crot | Santa Crux | sc-365976 | 1:500 |
| Runx2 (for IF) | Santa Cruz | sc-10758 | 1:1000 |
| Runx2 (for WB) | Abcam | ab236639 | 1:2000 |
| Rabbit IgG | Vector Laboratories | BA-1000 | 1:5000 |
| Mouse IgG | Vector Laboratories | BA-2000 | 1:5000 |
| Rabbit IgG | Thermo Fisher Scientific Inc. | A-11070 | 1:1000 |
| Mouse IgG | Thermo Fisher Scientific Inc. | A-21203 | 1:500 |

IF; immunofluorescence, WB; western blotting

**Cultured primary cells**

| Name | Vendor | Catalog # | Age, Sex, Race |
| --- | --- | --- | --- |
| Human coronary artery smooth muscle cells | PromoCells | C-12511 | 55, Male, Caucasian |
